# Adoption of Focus Groups in Designing Interventions to Address Vaccine Hesitancy Among Adolescents and Their Parents: A Systematic Review

**DOI:** 10.3390/vaccines13111108

**Published:** 2025-10-29

**Authors:** Patrizia Calella, Concetta Paola Pelullo, Gabriella Di Giuseppe, Francesco Napolitano, Fabrizio Liguori, Giorgio Liguori, Francesca Gallè

**Affiliations:** 1 Department of Medical, Movement and Wellbeing Sciences, University of Naples “Parthenope”, 80133 Naples, Italy; concettapaola.pelullo@uniparthenope.it (C.P.P.); giorgio.liguori@uniparthenope.it (G.L.); 2 Department of Experimental Medicine, University of Campania “Luigi Vanvitelli”, 80138 Naples, Italy; gabriella.digiuseppe@unicampania.it (G.D.G.); francesco.napolitano2@unicampania.it (F.N.); 3 Department of Economics and Legal Studies, University of Naples “Parthenope”, 80132 Naples, Italy; fabrizio.liguori@studenti.uniparthenope.it

**Keywords:** vaccine hesitancy, Adolescence, focus group, immunization

## Abstract

Background/Objectives: To address vaccine hesitancy, health promotion strategies must go beyond passive information delivery and identify individuals’ beliefs about vaccination. Focus groups (FGs) have emerged as promising tools in health education and behavioral change initiatives. Methods: To assess the employ of FGs in planning immunization strategies for adolescents, a systematic review of literature was performed from inception to July 2025 following PRISMA guidelines. PubMed/MEDLINE, Scopus, Web of Science, and Google Scholar databases were searched. Conclusions: Twenty articles met the inclusion criteria. In these studies, FGs were used to identify barriers or facilitators to vaccination, mainly to address HPV vaccine hesitancy. Across the WHO SAGE 3C framework, the outcomes of FGs clustered more in confidence and convenience/constraints than in complacency domain. These findings highlight the potential of FGs in designing programs to increase vaccine uptake. Further research on their possible role in motivating parents or adolescents towards immunization is needed.

## 1. Introduction

Vaccine hesitancy, defined as the “delay in acceptance or refusal of vaccines despite the availability of vaccination services”, represents a major threat to global health according to the World Health Organization (WHO) [1]. Numerous variables contribute to this complex and situation-specific phenomenon. According to MacDonald et al. [2], the main determinants can be grouped into three key domains—complacency, convenience, and confidence—which together form the so-called “3C model” of vaccine hesitancy. Although vaccination is essential for preventing infectious diseases, and it is one of the most economical and efficient public health measures, lowering morbidity and mortality, and fostering herd immunity at the community level, hesitancy keeps undermining public health initiatives and fuels the spread of diseases like measles and pertussis that may be prevented [3]. When vaccination acceptance is less than what would be predicted given the information and services offered, vaccine hesitancy is evident. It is a complicated and situation-specific phenomenon that changes with time, location, and vaccination in fact globally, vaccine hesitancy has shown variable prevalence [4]. According to a global systematic review conducted by Lane et al. (2018), hesitancy was reported in over 90% of WHO member states, with regional variations influenced by cultural, political, and socioeconomic contexts [5]. According to the “State of Vaccine Confidence in the European Union (EU) 2022” report, vaccine confidence across the EU remains generally high but uneven, with notable country-specific variations and concerning emerging trends. Between 2018 and 2020, public confidence in vaccines, particularly the influenza vaccine, significantly increased, likely driven by heightened risk perception during the onset of the COVID-19 pandemic. However, from 2020 to 2022, these gains have partly reversed, with overall perceptions of vaccine importance, safety, and effectiveness showing a decline, especially among younger adults aged 18–34 [6]. This widening intergenerational “vaccine confidence gap” raises concerns for the sustainability of immunization programs, as young adults are key for the uptake of routine childhood vaccines. Moreover, while healthcare professionals across the EU still display generally high confidence in vaccines, some countries, including France, Greece, and Slovakia, have reported recent declines even among this trusted group [6]. These findings underline the urgent need for sub-national monitoring and targeted interventions to prevent further erosion of trust.

There are many different and intricate factors that contribute to vaccine reluctance. According to the WHO SAGE Working Group, they are frequently divided into three categories: contextual factors (historical, cultural, and sociopolitical), individual and collective factors (beliefs, attitudes, and knowledge), and issues unique to vaccines and vaccinations (risk–benefit analysis, mode of administration) [2].

In adolescents, vaccine hesitancy can be influenced not only by personal beliefs but also by parental attitudes, peer groups, media exposure, and trust in healthcare providers. A recent study conducted in Italy, Portugal, Poland, and Spain found that vaccine hesitancy affects around 21% of both adolescents and parents, with significant variability between countries. Polish and Italian participants showed higher levels of hesitancy compared to Spanish counterparts. The most common reasons cited were fear of side effects and low trust in government recommendations [7]. Schools and educational institutions, as well as family dynamics, play a critical role in shaping adolescents’ understanding of and attitudes toward vaccination.

The COVID-19 pandemic has brought vaccine hesitancy into sharper focus. On one hand, the pandemic has stimulated increased awareness of vaccine science and accelerated public health communication efforts. On the other hand, it has also fueled skepticism, misinformation, and polarization, especially surrounding the rapid development and emergency approval of COVID-19 vaccines [8]. Several studies have documented rising levels of hesitancy, particularly in younger populations, due to perceived risks, mistrust in institutions, and the influence of social media [9,10]. For example, a longitudinal study by Paul et al. (2021) in the UK found that vaccine hesitancy among adolescents increased during the COVID-19 vaccination campaign, especially in regions with lower socioeconomic status and higher misinformation exposure [11].

To address vaccine hesitancy, health promotion strategies must go beyond passive information delivery and engage individuals in active, dialogic processes. In this context, focus groups (FGs) have emerged as promising tools in health education and behavioral change initiatives. An FG is a qualitative research technique that involves guided discussion among a small group of individuals to explore their perceptions, beliefs, and attitudes about a specific topic [12]. FG can be used in public health not only for exploratory research but also as instructional tools that encourage critical thinking and peer learning [13]. Particularly in vaccine communication, FG can allow participants to voice concerns, ask questions, and receive tailored responses in a supportive environment, potentially enhancing trust and acceptance [14].

In order to assess the current spread of FGs in immunization strategies planning, this review was aimed to systematically synthesize 1) the global evidence on their adoption in programs aimed to increase vaccine uptake and 2) barriers and facilitators to vaccination that emerge as FG outcomes.

## 2. Materials and Methods

This systematic review was conducted in accordance with the Preferred Reporting Items for Systematic Reviews and Meta-Analyses (PRISMA) guidelines (Supplementary Files S1 and S2). The protocol was prospectively registered with the International Prospective Register of Systematic Reviews (PROSPERO; registration number CRD420251111040). The review followed a predefined methodological approach to identify, select, and synthesize evidence on the effectiveness of FG-based interventions in reducing vaccine hesitancy among adolescents and adult populations.

### 2.1. Search Strategy

A comprehensive search strategy was implemented across the following electronic databases from inception to July 2025: PubMed/MEDLINE, Scopus, Web of Science, and Google Scholar. The search was designed to capture studies investigating the use of FG-based interventions aimed at improving vaccine knowledge, attitudes, or uptake. A combination of Medical Subject Headings (MeSH) and free-text terms was used to capture both interventional and qualitative/formative uses of FG among adolescents and/or parents/caregivers. Core terms included: “vaccine hesitancy”, “vaccination attitudes”, “vaccination uptake”, “intention to vaccinate”, “focus group*”, “group discussion*”, “community dialog*”, “workshop*”, “peer-led”, and population terms (“adolescent*”, “parent*”, “caregiver*”). To map formative applications, we added “barrier*”, “facilitat*”, “acceptab*”, “feasibil*”, “co-design”, “message testing”, and “formative”. Boolean operators (AND/OR) were applied appropriately, and MeSH terms (e.g., “Vaccination”, “Focus Groups”, “Adolescent”, “Parents”) were exploded where relevant. Search filters encompassed randomized, quasi-experimental, and pre–post designs, as well as qualitative, mixed-methods, and implementation/evaluation studies. Only human studies published between 2000 and 2025 in English or Italian language were considered. Additionally, reference lists of included articles and relevant reviews were hand-searched to identify any studies not retrieved through database searches. Search logs were maintained throughout the process.

### 2.2. Study Selection

Studies were eligible if they met the following criteria:

- Population: Adolescents (10–19 years) and/or their parents/caregivers.
- Concept: Studies that employed FG with adolescents and/or parents/caregivers in the context of vaccination, regardless of whether FG served a formative, message-testing, stakeholder-engagement, implementation/evaluation, or active interventional role.
- Comparator: Recorded if applicable (e.g., usual care, alternative education, or none in single-arm designs); a comparator was not required for inclusion.
- Outcomes: Reported at least one of the following: vaccine-related knowledge, attitudes, intention to vaccinate, actual uptake, and/or barriers and facilitators to vaccination (the latter mapped to the WHO SAGE 3C framework: confidence, complacency, convenience) [2]. Quantitative change was not required for qualitative studies.
- Study design: Randomized controlled trials, quasi-experimental, pre–post, qualitative, or mixed-methods designs.
- Setting: Any setting (healthcare, school, community, or online).
- Other limits: Human studies, published in English or Italian, from 2000 to 2025.

We excluded studies having adult individuals as immunization target; studies that did not involve FG as a clearly defined component; editorials, commentaries, conference abstracts, and literature reviews (except when used for reference screening); and studies not published in English or Italian.

Two independent reviewers (P.C. and F.L.) screened titles and abstracts to identify potentially relevant studies. Full texts of eligible articles were then assessed in detail. Disagreements between reviewers were resolved through discussion or consultation with a third reviewer (F.G.).

### 2.3. Data Extraction

Two reviewers (P.C. and F.N.) independently extracted data using a single, harmonized and pilot-tested form. For each study we captured: (a) bibliographic and contextual information (authors, year, country/region, setting); (b) population details restricted to adolescents and/or parents/caregivers (eligibility criteria, recruitment, sample size, key demographics); (c) study design and analytic approach (qualitative, mixed methods, pre–post, randomized/non-randomized); and (d) FG characteristics purpose/role within the study, question-guide focus, number and duration/frequency of sessions, group size/composition, facilitator background, delivery mode (in-person/online), and setting. Where reported, we also extracted implementation features (e.g., language or cultural adaptation, stakeholder involvement), and comparator (if applicable, e.g., usual care, alternative education, none).

Outcomes were extracted uniformly across studies: vaccine-related knowledge, attitudes, intention to vaccinate, actual uptake, and barriers/facilitators to vaccination. Barriers/facilitators were mapped to the WHO SAGE 3C framework (confidence, complacency, convenience) [2] and organized into contextual, individual/social, and vaccine/vaccination-specific domains; representative quotations were recorded when available. Equity-relevant variables (e.g., socioeconomic indicators, migration/language considerations, access/logistical constraints) were captured when reported. Prior to synthesis, all extracted records were rechecked for alignment with inclusion criteria and harmonized for consistent reporting.

Any disagreement between reviewers was resolved through discussion, with the involvement of a third reviewer (G.D.G.) when necessary. Post-extraction, records were rechecked for alignment with inclusion criteria and harmonized for reporting prior to synthesis (descriptive mapping and thematic synthesis).

### 2.4. Quality Assessment

We appraised study quality using the Mixed Methods Appraisal Tool (MMAT), applying a single framework across qualitative, quantitative (randomized, non-randomized, pre–post/descriptive), and mixed-methods designs. Two reviewers (P.C. and C.P.P.) independently judged each study against the five MMAT criteria relevant to its design category; disagreements were resolved by discussion or a third reviewer (F.G.). In line with MMAT guidance, we did not compute an overall numeric score; instead, we report criterion-level judgments (“Yes/No/Can’t tell”) per study and summarize patterns across the corpus [15].

## 3. Results

### 3.1. Study Selection

**Figure 1:**
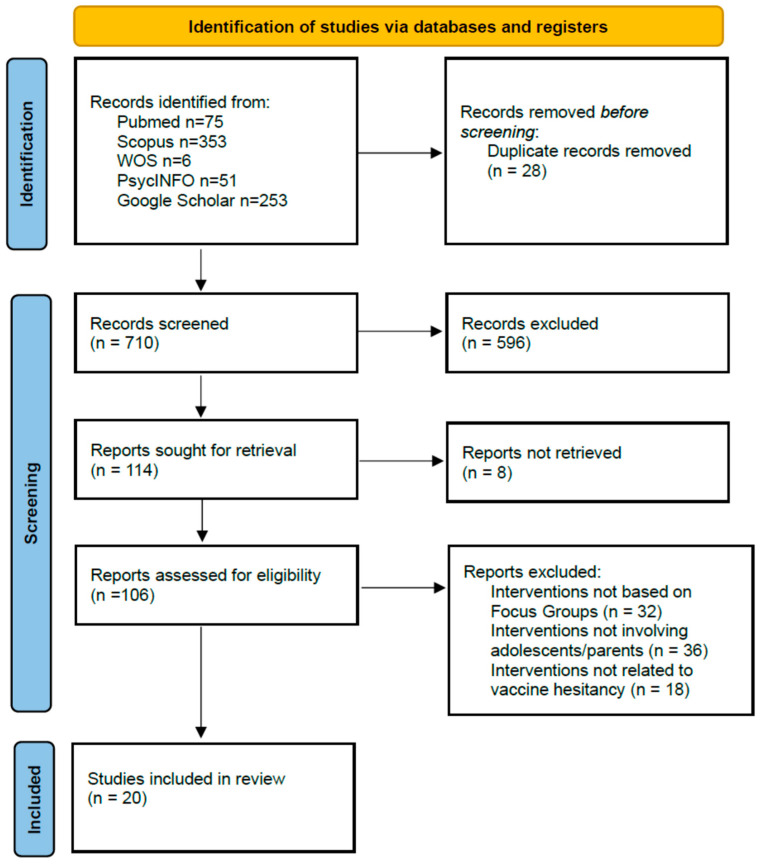
PRISMA flow diagram of literature search. Note: The difference in the number of records between Scopus and WoS databases reflects their specific coverage. Scopus indexes more books, book-chapters, reference books, and monographs when compared to WoS.

Figure 1 shows the flow diagram of the selection process. The database search yielded a total of 738 records, 28 of which were duplicates. After title and abstract screening, 106 studies were retained for full-text review. Of these, 77 were excluded for the following main reasons: no use of FG (n = 32), wrong population (adults only, n = 27), or not being related to vaccines (n = 18). Finally, 20 studies were included in the review.

### 3.2. Study Characteristics

A total of 20 studies met the inclusion criteria [16,17,18,19,20,21,22,23,24,25,26,27,28,29,30,31,32,33,34,35]. Study-level characteristics are summarized in Table 1. Publications spanned 2011–2025 (inclusive), covering a 14-year period. Studies were conducted in the United States (n = 10) [18,19,20,21,22,23,24,26,27,29]; South Africa (n = 3) [16,18,25]; Australia (n = 2) [17,35]; Sweden (n = 2) [30,32]; Canada (n = 2) [31,34]; Kenya (n = 1) [33]; Pakistan (n = 1) [28]; and Nigeria (n = 1) [16]. One multi-country study included sites in Argentina, Malaysia, South Korea, and Spain [25]. Overall, the included evidence covered research conducted across North America, Europe, Africa, Asia, and Oceania.

As for the type of vaccination, the majority of the studies (n = 14) investigated awareness, attitudes, hesitancy, communication strategies, and school-based programs regarding human papillomavirus (HPV) vaccine [17,18,19,20,22,23,26,27,28,29,31,32,33,34]. A smaller number of studies explored multicomponent adolescent vaccines, including tetanus–diphtheria–acellular pertussis (Tdap), quadrivalent meningococcal conjugate (MCV4/MenACWY), and HPV [21,24,30,35]. One study focused on influenza vaccination in school settings [19], while another addressed COVID-19 vaccine acceptability among adolescents and parents [16].

### 3.3. Study Participants

Across the 20 included studies, an estimated total of approximately 2200 participants took part in FG discussions. The size of the study samples varied considerably, ranging from small groups of about 20–30 participants [23,29] to large studies exceeding 120 participants [17,25].

Most studies involved parents/caregivers only (n = 11) [18,22,23,24,25,26,27,29,30,31,35], with mothers often being the primary participants. Reported parental age ranged from early adulthood (31 years) to midlife (up to 61 years), with specific studies targeting mothers of children aged 9–17 years [22,25,31]. One study also included parents of younger children (7–9 years) [34].

Three studies reported adolescents as the sole participants [16,28,32]. Both adolescents and their parents were included in 6 studies [17,19,20,21,33,34]. Some studies also incorporated perspectives from teachers, nurses, or healthcare providers in addition to adolescents or parents. Age ranges of adolescents most commonly fell between 10 and 19 years, with some studies specifying narrower brackets such as 11–18 years [20,21], 10–14 years [33], and 15–18 years [25]. A few studies included slightly older youth, up to 24 years of age [16].

### 3.4. Focus Group Characteristics

FGs were most commonly organized in community settings [18,22,23,24,25], but they were also frequently embedded in school environments [17,19,21,30,32]. Other locations included clinics or health centers [20,26,31,33], child healthcare centers [34], or healthcare facilities together with schools and communities [16,35]. Detailed parameters of FG delivery (number of groups, typical size, and duration) are reported in Table 1.

Across the included studies, a total of approximately 159 FG were conducted, with an average of 8–9 groups per study. The number of groups per study ranged widely from as few as 2–3 sessions [21,23] to more extensive designs with over a dozen groups [17,25]. Most studies conducted a single session per participant, although a minority included follow-up discussions. When reported, session length typically ranged from 60 to 120 min [21,26,29], with an overall average of approximately 67 min. The majority of FG were held in-person [17,18,19,20,22]. However, several more recent studies adopted online or hybrid formats, reflecting the expansion of virtual engagement during and after the COVID-19 pandemic [26,27,29,34]. FG were predominantly moderated by trained researchers or project staff, often with specific expertise in qualitative methods [20,26]. Some studies highlighted the use of bilingual facilitators to ensure cultural and linguistic congruence, particularly in immigrant or minority populations [22,23]. In other cases, public health professionals or community partners played a central role in leading sessions, especially in applied and intervention-oriented contexts [16,21,35].

All included studies (n = 20) used focus groups primarily to map barriers and facilitators, probing perceptions of vaccine benefits/risks, procedural pain, school/clinic logistics, cultural–linguistic obstacles, and trust/mistrust dynamics [16,17,18,19,20,21,22,23,24,25,26,27,28,29,30,31,32,33,34,35]. Stakeholder engagement, with FGs involving parents, adolescents, and community partners to refine messaging, identify trusted messengers, and adapt delivery pathways, was also common (n = 10), refs [16,24,27,28,29,30,31,33,34,35]. Message testing for tone, clarity, cultural/linguistic congruence, and channel preferences was one of the aims of FGs in 8 studies [21,22,26,27,28,30,31,34]. True co-design processes (e.g., collaborative prototyping of materials) were reported in only one study [26]. Finally, FG was used to troubleshoot delivery constraints or inform service pathways during roll-out in one study [24].

### 3.5. Cross-Study Synthesis of Focus-Group Findings

Across settings, baseline HPV knowledge was frequently limited and interlaced with misconceptions (e.g., timing before sexual debut; need for boys), prompting requests for clear, age-appropriate explanations and rationale. This pattern emerged among parents and adolescents in the USA and Canada, and among adolescents/parents in Pakistan and Kenya, with cultural and language tailoring repeatedly flagged as essential [21,28,31,33]. Children themselves asked for simple, trusted explanations delivered directly to them, not only to parents [32].

Mothers often led day-to-day decisions, but other family figures (e.g., fathers, grandmothers) were pivotal gatekeepers in several contexts; adolescents sometimes initiated vaccine discussions with parents (school-based influenza exemplar; HPV spillover) [18,19,28]. Parents of youth with disability showed a wide spectrum from pro-vaccine to hesitant, often expressing heightened protectiveness and distinct concerns (e.g., needle trauma, assumptions about sexual inactivity) that require tailored engagement [35]. Parents favored concise, culturally/linguistically adapted materials and decision aids that directly address their concrete questions (e.g., safety, side effects, rationale for vaccinating boys, dose schedules) [21,31]. Parents valued authentic stories from other parents when these aligned with scientific evidence; however, negative anecdotes were more salient online, increasing the need for credible, verifiable narratives and moderation on social platforms [27].

Fear, pain, and peer contagion of distress were salient among school-vaccinated adolescents and could disrupt delivery; proactive management (preparation, distraction techniques, supportive peer climate, scheduling) mitigated these effects [17]. Framing HPV vaccine as a routine adolescent vaccine and emphasizing cancer prevention helped normalize uptake and address timing misconceptions; segment-specific tailoring (e.g., “undecided/ready if offered” vs. “undecided/skeptical”) improved relevance [20].

Practical barriers (transport, work schedules, consent complexity, language access) and clinic experiences affected uptake, especially in LMIC or underserved settings; suggested solutions included simplified consent, translated materials, household visits, community camps, and clear written take-home information for parents [19,28,33]. Where implemented, school-based immunization remains a key equity lever, but participants recommended formalized curricula (Grade 6) co-delivered by nurses and teachers, with student-centered content and parent-directed components [34].

Parents and providers across five countries expressed preference for 2-dose HPV regimens (≤15 years) due to lower cost, fewer visits, and better completion, while seeking assurance on safety/efficacy highlighting how program logistics and evidence communication intersect [25]. The broader post-pandemic vaccine discourse (fatigue, generalized distrust) spilled into adolescent vaccination conversations in some settings, especially among youth [16,35].

### 3.6. Barriers and Facilitators Mapped to the WHO SAGE 3C

Across the 20 studies, most barriers clustered under *Confidence*, including safety/side-effect concerns, misinformation, and institutional mistrust [16,23,26,27,29]. Facilitators included a strong provider recommendation, trusted nurse–family relationships, clear age-appropriate rationales, and framing HPV as routine/cancer-prevention [20,21,22,30,31].

*Complacency* reflected low perceived risk or delayed need (e.g., assumptions about sexual inactivity); facilitators included explicit risk communication, school-grade curricula, and adolescent-centered explanations [18,32,34].

*Convenience/constraints* encompassed consent complexity, language and access barriers, transport or work schedules, clinic experience, and fear during school delivery; facilitators included translated materials, simplified consent, school-based delivery, distraction techniques, written take-home information, and preference for 2-dose regimens ≤15 years [17,19,21,22,25,28,33,34].

A study-level summary of the primary 3C domain(s) and exemplar quotes is provided in Table 2.

### 3.7. Quality Appraisal

Overall, the quality was acceptable to good across the 20 studies. All studies clearly stated their research questions and collected data that addressed them. Among the 16 qualitative studies, most showed an appropriate qualitative approach, adequate data collection methods. A common limitation was partial coherence across data sources, analysis and interpretation, as well as incomplete reporting of sampling strategies and analytic procedures.

The four mixed-methods studies generally provided a sound rationale for combining methods and partially achieved effective integration of qualitative and quantitative components. However, only few explicitly addressed divergences between data sources. A detailed appraisal of each study is provided in Supplementary Table S1.

## 4. Discussion

The literature analyzed in this review highlights the potential of focus groups as a tool for interventions aimed at increasing vaccine acceptance among adolescents and their parents.

In particular, the selected studies employed focus groups to identify barriers or facilitators to vaccination. Identifying specific drivers of vaccine hesitancy is crucial to design targeted interventions [36]. This enables us to implement tailored strategies to overcome resistance, enhance vaccination uptake and improve resource allocation.

Allowing us to capture participants’ views and experiences, together with new issues and outlying opinion, FGs can represent a useful tool in public health strategy planning, if adequately guided [37]. However, the limited number of FG-based studies found in the literature with regard to vaccine hesitancy testifies a low adoption of this tool in identifying drivers of vaccine uptake among adolescents or their parents. It should be considered that FGs have some disadvantages, such as the possibility that participants conform to the majority opinions, the low generalization of the results and mainly the costs in terms of human resources, all factors that may hinder their adoption and probably contributed to the exiguity of evidence [37].

Though FGs can be useful also in addressing participants’ concerns regarding specific issues, to our knowledge no studies reported their adoption with this aim so far. This possible role deserves further investigation.

Across the included studies, focus groups were used mainly to map barriers and facilitators to vaccination. We used the WHO 3C model for this review because it is concise and practical; it categorizes vaccine-hesitancy drivers into three categories: confidence, complacency, and convenience/constraints. This allows for consistent synthesis and the design of focused interventions.

The 3C framework is widely applied in adolescent and HPV vaccination research [38] and in COVID-19 hesitancy studies making our results comparable to prior work. Crucially, it bridges focus-group insights to program levers, turning qualitative themes into concrete actions.

These three challenges are common to qualitative research that has employed focus groups outside of our corpus. Confidence-related issues dominate parents and adolescents grapple with conflicting information, fear of adverse effects, and variable trust in health services and policy makers; this is a recurrent theme in focus-group syntheses and qualitative reviews on HPV programs in high-income countries and has remained remarkably stable over the past decade [39]. These accounts mirror Holman and colleagues’ systematic review of barriers to adolescent HPV vaccination, which highlights safety worries, doubts about necessity, and provider-side constraints as persistent impediments [40]. Focus groups with young women in Malaysia highlight low baseline knowledge, strong social norms, and safety concerns, mirroring the confidence and social-process drivers seen in our review [41].

Complacency also plays a visible role in FG narratives. Parents often perceive low near-term risk and question the timing of HPV vaccination before sexual debut, while adolescents themselves report limited baseline knowledge and ambivalent social norms. Such patterns are echoed in qualitative syntheses of parental decision-making and in school-based studies in the United Kingdom, where attitudes shift when rationales are explained clearly and endorsed by trusted practitioners [39]. Online focus groups with vaccine-refusing parents in the Netherlands emphasize distrust of authorities, safety concerns, and practical hurdles, reinforcing confidence and constraint domains [42].

Constraints complete the picture. FG-based work with migrant and refugee families in Europe describes practical barriers that complicate otherwise favorable intentions: language hurdles, navigation of entitlements, appointment logistics, and transport or work schedules. Systematic reviews of migrants confirm these themes and point to the need for tailored, low-barrier pathways that close the gap between intention and uptake [43,44].

Finally, school delivery introduces its own texture of barriers and solutions that FG participants describe vividly. Observational and focus-group studies in Australian school programs document fear, pain, privacy concerns, and “peer contagion” of distress during vaccination sessions, suggesting that procedural and environmental adjustments are integral to equity and acceptability. Pragmatic evaluations of the CARD approach in Canadian schools show that structured, nurse-led strategies that prepare students, reorganize clinic flow, and normalize coping behaviors can improve the vaccination experience at scale [45].

Our findings fit together with the review by Jarrett et al., which concluded that multicomponent, dialog-based approaches are most effective for addressing vaccine hesitancy, with moderate-quality evidence supporting social mobilization, mass-media campaigns, communication training for healthcare workers, and reminder/recall systems [46]. Given that all included studies in our review used FGs to surface contextual barriers and facilitators, our results provide the formative substrate that such strategies require. At the same time, Jarrett et al. noted the scarcity of robust impact evaluations, underscoring the need for prospective studies that link FG-informed adaptations to downstream motivation and uptake [46].

As for the context, it should be noted that the majority of the selected studies used FGs in designing communication interventions to address hesitancy towards HPV vaccination. This reflects the higher need to implement programs aimed at increasing HPV vaccine uptake than the others. In fact, evidence shows that barriers to HPV vaccination may differ from that to other vaccinations, which commonly include fear of side effects, misinformation and mistrust in healthcare system, and include also low awareness of HPV-related cancer risks, and cultural stigma associated with a sexually transmitted infection [29,47,48]. The limited number of studies performed on influenza vaccination, despite its global importance in controlling annual pandemics, highlights a research gap. This is probably due to the lower perceived burden of influenza among adolescents or to the lack of resources that can prioritize vaccines with more polarized public debate (e.g., HPV, COVID-19).

Taken together, these insights reinforce one concept: in order to move the needle on adolescent vaccination, programs must pair credible, developmentally appropriate communication and unequivocal clinical recommendations with practical redesign of delivery, particularly in schools and in communities where language, access, or trust are fragile. FGs can represent a crucial tool in this direction.

This review has some limitations. First, the search and selection procedures were limited to the literature in English and Italian language. This could have generated a selection bias, possibly excluding other FG-based interventions that involved populations from other countries than those reported in the review. Moreover, given the variability of the interventions and the qualitative nature of the outcomes examined, it was not possible to further compare the results of the studies. With the expected growing of literature in this field, it will be possible in the future to perform meta-analyses on the results coming from, for example, studies focused on a single type of vaccine. Finally, it should be considered that this review was focused on adolescents’ immunization. Given the different efficacy that communication strategies for vaccine hesitancy can have between different population groups [36], exploring the evidence regarding the use of focus groups in adults in the perspective of increasing their own vaccine uptake is needed to complement our findings.

Beyond the research context, translating FG-derived insights into actionable strategies requires institutional engagement. National and regional health authorities play a key role in integrating participatory approaches into vaccination programs, ensuring that qualitative evidence informs policy design and measurable public health actions. Recent policy analyses, such as those of Correia et al., underscore the need for structured collaboration between researchers, healthcare organizations, and decision-makers to bridge the gap between evidence generation and implementation in vaccine hesitancy strategies [49].

## 5. Conclusions

The findings of this review support using FGs upstream in program planning to tailor demand-side strategies and to adapt workflows to local constraints. Future research should move beyond formative mapping and prospectively link FG-informed adaptations to motivation, initiation, and series completion, with equity-sensitive evaluation and standardized reporting to enable comparison across vaccines and contexts. FG-based interventions with appropriate sample selection and evaluation of knowledge, intention, and vaccine uptake are needed. Finally, effective translation of FG-based evidence into vaccination practice requires strong collaboration with national and regional health authorities to ensure that participatory approaches inform policy design and measurable public health actions.

## Figures and Tables

**Table 1:** Characteristics of included studies.

First Author (Year)	Country	Vaccine(s)	Population (A/P/Both); n Total	Setting	Design	FG Sessions (No. Groups; Participants Group; Duration)	FG Role
Bernard (2011) [17]	Australia (NSW)	HPV	A; 120	School	Qualitative (FG + obs + interviews)	20; 6–10; ~60–90′	Evaluation
Francis & Katz (2013) [18]	South Africa; USA (Ohio Appalachia)	HPV	P; SA n = 24, USA n = 19	Community	Qualitative (FG)	SA: 3; USA: 6; 60–90′	Formative
Herbert (2013) [19]	USA (Georgia)	Influenza	Both; NR	Community (linked to school clinics)	Qualitative (FG)	8; NR; NR	Evaluation
Hull (2014) [20]	USA	HPV	Both; NR	Community	Qualitative (FG + interviews)	8; mothers 3–7 / daughters 3–9; NR	Formative
Greenfield (2015) [21]	USA (King County)	HPV/MCV4/ Tdap	P (mothers); NR	Community/Clinics/School-based HC	Mixed (surveys + FG)	3; NR; NR	Formative
Valdez (2015) [22]	USA (California)	HPV	P; 83 (42 Latino, 41 Korean)	Community organizations	Formative qual (FG + cognitive int.) + RCT DVD	6; 13–15; NR	Formative
Albright (2017) [23]	USA (Colorado)	HPV	P; 11	Public community sites	Qualitative (FG + IDI)	2; NR; NR	Formative
Schoeppe (2017) [24]	USA (Washington State)	HPV/MCV4/ Tdap	P; NR	Schools and Community	Mixed-methods evaluation (FG + interviews + surveys)	NR; NR; NR	Evaluation
Islam (2018) [25]	Argentina, Malaysia, South Africa, South Korea, Spain	HPV	P (mothers); 124	Medical offices/Health centers/Schools	Mixed-methods (provider surveys + FG/IDI)	16; 5–6; NR	Evaluation
Becker (2021) [26]	USA (Texas)	HPV	P; NR	Pediatric clinic network	Qualitative (text-based online FG)	4; 4–7; NR	Formative
Massey (2021) [27]	USA	HPV	P; 48	Online (Twitter-based)	Qualitative (online FG)	6; 8; NR	Formative
Ali (2022) [28]	Pakistan (Sindh)	HPV	A (girls); 12	Schools and Community health facilities	Qualitative exploratory (FG)	4; NR; NR	Formative
Shin (2023) [29]	USA (Los Angeles)	HPV	P; NR	Online (Zoom)	Qualitative (FG)	4; 3–7; NR	Formative
Appelqvist (2023) [30]	Sweden	Childhood (incl. HPV)	P; 47	Child Health Centers and Schools	Qualitative (FG)	6; 4–11; NR	Evaluation
Dionne (2023) [31]	Canada (Quebec)	HPV	P; 22	School health services	Qualitative (FG + interviews)	3; 7–8; NR	Formative
Enskär (2024) [32]	Sweden	HPV	A (children 10–11); NR	School health services	Qualitative (FG)	6; 4–12; NR	Evaluation
Ochomo (2024) [33]	Kenya (Kisumu)	HPV	Both; 48	Hospital	Qualitative (FG)	4; 8; NR	Evaluation
Brohman (2024) [34]	Canada (British Columbia)	HPV	Both; NR (students + adults)	Schools	Qualitative (FG students + interviews adults)	7; 6–8 (students); NR	Formative/Evaluation
Carter (2024) [35]	Australia	HPV/MCV4/ Tdap	P + school staff + providers; 40	Special schools	Qualitative (FG + interviews)	NR; NR; NR	Formative
Casale (2025) [16]	South Africa; Nigeria	COVID-19	A/young people; FG n = 127 (SA) + IDI	CBO sites; virtual	Qualitative exploratory (FG + IDI)	12; NR; Online	Evaluation

Abbreviations: A = adolescents; P = parents; Both = mixed groups; Qual = qualitative; FG = focus group(s); obs = observations; IDI = in-depth interviews; HC = health center; NR = not reported; RCT = randomized controlled trial.

**Table 2:** Barriers and facilitators mapped to the WHO SAGE 3C framework across included studies.

Study (Country; Vaccine; Population)	Primary 3C Domain(s)	Key Barriers	Key Facilitators	Implications
Bernard 2011 (Australia; HPV; A) [17]	Convenience	Fear/pain; peer contagion; clinic environment	Nurse preparation; distraction; scheduling tweaks	Prepare students; manage pain/anxiety; optimize school flow
Francis & Katz 2013 (SA/USA; HPV; P) [18]	Confidence; Complacency; Convenience	Low awareness; safety/cost concerns; moral concerns	Culturally tailored education; family decision support	Tailored materials; address cost; engage mothers/grandmothers
Herbert 2013 (USA; Influenza; Both) [19]	Convenience; Confidence	Consent complexity; mistrust	Live Q&A; clear brochures	Simplify consent; add interactive communication
Hull 2014 (USA; HPV; Both) [20]	Confidence; Complacency	Safety/timing concerns; skepticism	Cancer-prevention framing; trusted recommendation	Normalize as routine; address timing before sexual debut
Greenfield 2015 (USA; HPV/MCV4/Tdap; P) [21]	Convenience; Confidence	Language barriers; limited HCP recommendation	Translated materials; strong provider advice	Provide translated decision aids; cue clinician recommendation
Valdez 2015 (USA; HPV; P) [22]	Confidence	Low knowledge; decisional conflict	Multilingual, culturally tailored DVD	Deliver tailored multimedia; reduce decisional conflict
Albright 2017 (USA; HPV; P) [23]	Confidence; Convenience	Safety/distrust; series logistics unclear	Language-specific strategies; series explanation	Segment by language; clarify series/visits
Schoeppe 2017 (USA; HPV/MCV4/Tdap; P) [24]	Confidence	Hesitancy despite information	Peer/community engagement	Use parent advocates; community-based messaging
Islam 2018 (Multi-country; HPV; P) [25]	Convenience; Confidence	Cost/visits; safety/efficacy concerns	Preference for 2-dose ≤15y	Adopt 2-dose where eligible; emphasize effectiveness
Becker 2021 (USA; HPV; P) [26]	Confidence	Misinformation; safety concerns	Pediatrician recommendation; tailored digital tools	Send pre/post-visit digital nudges; debunk myths
Massey 2021 (USA; HPV; P) [27]	Confidence	Negative anecdotes dominant online	Credible peer narratives; moderation	Verifiable stories; moderate social platforms
Ali 2022 (Pakistan; HPV; A) [28]	Confidence; Convenience	Low knowledge; family decision gatekeepers	Household visits; community camps	Engage fathers; bring services to communities
Shin 2023 (USA; HPV; P) [29]	Confidence; Convenience	Mistrust; logistical obstacles	Multilevel, tailored messaging	Address histories of mistrust; reduce access barriers
Appelqvist 2023 (Sweden; child vacc incl. HPV; P) [30]	Confidence	Safety/compliance questions (age-specific)	Nurse–family trust; tailored info by age	Empower nurses; age-adjusted counseling
Dionne 2023 (Canada; HPV; P) [31]	Confidence	Age appropriateness; side effects; boys; COVID interactions	Decision aids; social media tools	Provide concise decision aids
Enskär 2024 (Sweden; HPV; A 10–11) [32]	Confidence; Convenience	Need for clear reasons; fear/pain	School nurse as trusted expert; preparation	Explain early age and boys; manage procedural anxiety
Ochomo 2024 (Kenya; HPV; Both) [33]	Confidence; Convenience	Myths; negative clinic experiences	Written materials; parent involvement; community engagement	Provide translated take-home info; improve clinic experience
Brohman 2024 (Canada; HPV; Both) [34]	Confidence; Convenience	Missed education in SBIP; anxiety/pain	Grade-6 curriculum; parent education	Co-deliver nurse/teacher curricula; include pain/anxiety content
Carter 2024 (Australia; HPV/MCV4/Tdap; P + staff) [35]	Confidence; Convenience	Autism myths; trauma concerns; COVID fatigue	Tailored communication; adjustments	Counter myths; adapt procedures for special schools
Casale 2025 (SA/Nigeria; COVID-19; youth) [16]	Confidence; Complacency; Convenience	Misinformation; distrust; fatigue	Safety/efficacy beliefs; protection/normality	Rebuild trust; youth-friendly messages; address access

Abbreviations: 3C = Confidence, Complacency, Convenience; A = adolescents; P = parents; SBIP = school-based immunization program; HCP = healthcare provider; NR = not reported.

## Data Availability

No new data were created or analyzed in this study. Data sharing is not applicable to this article.

## Supplementary Materials

The following supporting information can be downloaded at: https://www.mdpi.com/article/10.3390/vaccines13111108/s1, Supplementary Table S1: MMAT quality assessment of included studies. Supplementary File S1, PRISMA_2020_abstract_checklist [50]; Supplementary File S2, PRISMA_2020_checklist [50].

## Abbreviations

The following abbreviations are used in this manuscript:

FG	Focus Group
MMAT	Mixed Methods Appraisal Tool
PRISMA	Preferred Reporting Items for Systematic Reviews and Meta-Analyses
PROSPERO	International Prospective Register of Systematic Reviews
