# Supplementary material for: Adoption of Focus Groups in Designing Interventions to Address Vaccine Hesitancy Among Adolescents and Their Parents: A Systematic Review
Source: Vaccines (Basel). 2025 Oct 29;13(11):1108. doi: 10.3390/vaccines13111108 (PMC12656320; doi:10.3390/vaccines13111108)
Supplement: Supplementary file 1 [file vaccines-13-01108-s001.zip › vaccines-3934395-Table S1.pdf]

Supplementary Table S1. Quality assessment MMAT of included studies

Fill codes: Y = Yes; N = No; CT = Can't tell.

Author (year)	Design	MMAT items (S1–S2; 1.1–1.5 / 5.1–5.5)	Brief comment
**Bernard et al., 2011 [17]**	Qualitative	S1:Y; S2:Y; 1.1:Y; 1.2:N; 1.3:Y; 1.4:Y; 1.5:CT	Rich school FG data; small sample; potential observation bias.
**Francis & Katz, 2013 [18]**	Qualitative	S1:Y; S2:Y; 1.1:Y; 1.2:CT; 1.3:Y; 1.4:CT; 1.5:CT	Cross-cultural; analysis detail limited;<br>cultural context strong.
**Herbert et al., 2013 [19]**	Qualitative	S1:Y; S2:Y; 1.1:Y; 1.2:Y; 1.3:Y; 1.4:Y; 1.5:CT	Parents/students; good coherence; partial sampling description.
**Hull et al., 2014 [20]**	Qualitative	S1:Y; S2:Y; 1.1:Y; 1.2:Y; 1.3:N; 1.4:Y; 1.5:Y	Participatory approach; solid analysis;<br>limited generalisability.
**Greenfield et al., 2015 [21]**	Mixed methods	S1:Y; S2:Y; 5.1:CT; 5.2:Y; 5.3:N; 5.4:CT; 5.5:Y	Survey + FGs; integration adequate; rationale MM not explicit.
**Valdez et al., 2015 [22]**	Mixed methods	S1:Y; S2:Y; 5.1:Y; 5.2:N; 5.3:Y; 5.4:CT; 5.5:CT	FGs + RCT; integration clear; qual analytic detail weak.
**Albright et al., 2017 [23]**	Qualitative	S1:Y; S2:Y; 1.1:Y; 1.2:Y; 1.3:Y; 1.4:CT; 1.5:CT	FGs+IDIs; bilingual; missing context detail; some bias risk.
**Schoeppe et al., 2017 [24]**	Mixed methods	S1:Y; S2:Y; 5.1:Y; 5.2:CT; 5.3:Y; 5.4:CT; 5.5:CT	Community engagement; integration partial; divergence unaddressed.
**Islam et al., 2019 [25]**	Mixed methods	S1:Y; S2:Y; 5.1:Y; 5.2:Y; 5.3:Y; 5.4:Y; 5.5:Y	Cross-country survey + FG; robust design; variation in reporting.
**Becker et al., 2021 [26]**	Qualitative	S1:Y; S2:Y; 1.1:Y; 1.2:N; 1.3:N; 1.4:Y; 1.5:CT	Online FGs; strong linkage data-findings; single-site context.
**Massey et al., 2021 [27]**	Qualitative	S1:Y; S2:Y; 1.1:Y; 1.2:Y; 1.3:Y; 1.4:Y; 1.5:Y	Parent social-media FGs; credible insights; limited transferability.
**Ali et al., 2022 [28]**	Qualitative	S1:Y; S2:Y; 1.1:Y; 1.2:N; 1.3:Y; 1.4:CT; 1.5:CT	Girls' recommendations; rich context; partial analytic depth.
**Appelqvist et al., 2023 [30]**	Qualitative	S1:Y; S2:Y; 1.1:Y; 1.2:Y; 1.3:Y; 1.4:Y; 1.5:CT	Parental views; appropriate methods; coherence partial.
**Dionne et al., 2023 [31]**	Qualitative	S1:Y; S2:Y; 1.1:Y; 1.2:Y; 1.3:N; 1.4:Y; 1.5:Y	Parents + school nurses; strong thematic analysis.
**Shin et al., 2023 [29]**	Qualitative	S1:Y; S2:Y; 1.1:Y; 1.2:Y; 1.3:CT; 1.4:CT; 1.5:CT	Minority parents; multilevel lens; interpretation somewhat weak.
**Brohman et al., 2024 [34]**	Qualitative	S1:Y; S2:Y; 1.1:Y; 1.2:CT; 1.3:Y; 1.4:Y; 1.5:CT	Students/parents/staff; robust; partial coherence across sources.
**Carter et al., 2024 [35]**	Qualitative	S1:Y; S2:Y; 1.1:Y; 1.2:Y; 1.3:CT; 1.4:CT; 1.5:CT	Special schools; diverse stakeholders; analytic limits.

**Enskär et al., 2024 [32]**	Qualitative	S1:Y; S2:Y; 1.1:Y; 1.2:Y; 1.3:Y; 1.4:Y; 1.5:Y	Children's perspective; clear coherence and substantiation.
**Ochomo et al., 2024 [33]**	Qualitative	S1:Y; S2:Y; 1.1:Y; 1.2:CT; 1.3:Y; 1.4:CT; 1.5:CT	Kenyan adolescents/parents; insights on myths; limited detail on analysis.
**Casale et al., 2025 [16]**	Qualitative	S1:Y; S2:Y; 1.1:Y; 1.2:Y; 1.3:Y; 1.4:Y; 1.5:CT	Youth COVID-19 acceptability; mixed data sources; limited transferability.

Abbreviations

Y = Yes; N = No; CT = Can't tell; RQs = Research questions; MM = Mixed methods; FGs = Focus groups; IDIs = In-depth interviews.
